# A Mobile Prenatal Care App to Reduce In-Person Visits: Prospective Controlled Trial

**DOI:** 10.2196/10520

**Published:** 2019-05-01

**Authors:** Kathryn I Marko, Nihar Ganju, Jill M Krapf, Nancy D Gaba, James A Brown, Joshua J Benham, Julia Oh, Lorna M Richards, Andrew C Meltzer

**Affiliations:** 1 The George Washington University School of Medicine and Health Sciences Washington, DC United States; 2 OB Hospitalist Group Baylor All-Saints Medical Center Fort Worth, TX United States; 3 University of Pittsburgh School of Medicine Pittsburgh, PA United States; 4 The Jackson Laboratory Farmington, CT United States; 5 Department of Emergency Medicine George Washington University School of Medicine and Health Sciences Washington, DC United States

**Keywords:** prenatal care, mobile applications, patient monitoring, patient safety, patient satisfaction, technological innovations, controlled clinical trial, mobile health

## Abstract

**Background:**

Risk-appropriate prenatal care has been asserted as a way for the cost-effective delivery of prenatal care. A virtual care model for prenatal care has the potential to provide patient-tailored, risk-appropriate prenatal educational content and may facilitate vital sign and weight monitoring between visits. Previous studies have demonstrated a safe reduction in the frequency of in-person prenatal care visits among low-risk patients but have noted a reduction in patient satisfaction.

**Objective:**

The primary objective of this study was to test the effectiveness of a mobile prenatal care app to facilitate a reduced in-person visit schedule for low-risk pregnancies while maintaining patient and provider satisfaction.

**Methods:**

This controlled trial compared a control group receiving usual care with an experimental group receiving usual prenatal care and using a mobile prenatal care app. The experimental group had a planned reduction in the frequency of in-person office visits, whereas the control group had the usual number of visits. The trial was conducted at 2 diverse outpatient obstetric (OB) practices that are part of a single academic center in Washington, DC, United States. Women were eligible for enrollment if they presented to care in the first trimester, were aged between 18 and 40 years, had a confirmed desired pregnancy, were not considered *high-risk*, and had an iOS or Android smartphone that they used regularly. We measured the effectiveness of a virtual care platform for prenatal care via the following measured outcomes: the number of in-person OB visits during pregnancy and patient satisfaction with prenatal care.

**Results:**

A total of 88 patients were enrolled in the study, 47 in the experimental group and 41 in the control group. For patients in the experimental group, the average number of in-person OB visits during pregnancy was 7.8 and the average number in the control group was 10.2 (*P*=.01). There was no statistical difference in patient satisfaction (*P*>.05) or provider satisfaction (*P*>.05) in either group.

**Conclusions:**

The use of a mobile prenatal care app was associated with reduced in-person visits, and there was no reduction in patient or provider satisfaction.

**Trial Registration:**

ClinicalTrials.gov NCT02914301; https://clinicaltrials.gov/ct2/show/NCT02914301 (Archived by WebCite at http://www.webcitation.org/76S55M517)

## Introduction

### Background

In the United States, there are nearly 4 million live births each year. This makes prenatal care one of the most widely utilized preventative care health services [1,2]. Despite its widespread use, the effectiveness and organization of standard prenatal care has been debated [3-5]. Rigorous scientific evidence of the effectiveness of standard prenatal care, including effects on maternal and infant outcomes, health-related behaviors, and health care costs is limited [2]. In the early mid-1980s, an expert panel recommended stratifying women into high- and low-risk categories, with high-risk women receiving more intensive prenatal care and low-risk women following a reduced visit frequency schedule [6]. The rationale for this recommendation was that unnecessary visits for low-risk patients consume health care resources that could be applied more judiciously to women with higher-risk pregnancies. However, despite these recommendations, the standard model of prenatal care with high-frequency visits has persisted. Almost a third of low-risk women receive more visits than recommended by the American College of Obstetricians and Gynecologists (ACOG) [7]. The barriers cited by providers for not reducing visits for low-risk pregnancies include the following: concern about decreased patient satisfaction, need for frequent weight and blood pressure monitoring, and concern about reduced transmission of educational information regarding health and lifestyle choices during pregnancy.

### Goal of This Study

The primary objective of this study was to determine if a mobile prenatal care app facilitates a reduced in-person prenatal care visit schedule while maintaining patient and provider satisfaction. Mobile health apps have the potential to address many of the perceived barriers to reducing in-person visits [8]. The application of mobile phone technology has been shown to improve disease management for diabetes self-care activities, HIV infection medication adherence, and sickle cell anemia medication adherence [9-11]. We hypothesize that a similar approach using a mobile app and connected monitoring devices may also be beneficial to manage prenatal care. A pilot trial with 8 patients was performed. This trial ensured the platform and devices functioned appropriately, any blood pressure and weight triggers were identified and managed, a reduced visit schedule was achieved on a small scale, and the platform was satisfactory to patients. The feedback from the pilot guided this investigation [12].

## Methods

### Study Design

This pragmatic controlled trial compared an experimental group that received a mobile app for prenatal care and an integrated Wi-Fi-connected blood pressure and weight scale with a control group that received usual care. Institutional Review Board (IRB) approval has been obtained from the George Washington University (GWU) IRB (IRB#: 015422).

### Study Setting

The educational components and clinical triggers were developed and refined at GWU in Washington, DC, United States, working in conjunction with a local mobile health technology firm 1EQ and their product Babyscripts [13]. The clinical trial occurred in 2 obstetric (OB) and gynecology (GYN) offices in the United States: one in downtown Washington, DC, and one in suburban Maryland. Prenatal care is provided at both locations by OB and GYN physicians and nurse midwives. Low-risk women are cared for by obstetrician-gynecologists at both locations, and all deliveries take place at the GWU hospital in Washington, DC. Enrollment occurred between July 2015 and March 2016.

### Inclusion and Exclusion Criteria

Eligible participants were women aged between 18 and 40 years, presenting for a first trimester (up to 13 weeks gestational age) verification of pregnancy or new OB visit, and who were considered low-risk. Low-risk was defined as a singleton pregnancy with no previous diagnosis of essential hypertension, diabetes, renal disease, collagen vascular disease, maternal substance abuse, or other previously documented condition posing a high risk of poor pregnancy outcome. Dropout criteria for the study included the antepartum diagnosis of fetal abnormalities, placenta previa, intrauterine growth restriction, pregnancy-induced hypertension, gestational diabetes, or premature rupture of membranes. (Multimedia Appendix 1) Participants were also required to regularly use a mobile phone and be fluent in English.

### Allocation

Allocation into the experimental group versus the control group was based on the operating system of the patient’s mobile phone. Patients with iOS-based mobile phones (ie, iPhones) were allocated to the experimental group; patients with Android or Windows operating systems were allocated to the control group. This allocation system was chosen as a practical solution to the challenges of randomization and blinding. In addition, the app had yet to be developed on the Android platform. Allocation was concealed until after consent was obtained.

**Table 1:** Sample alternative prenatal care schedules.

Schedule	New OB^a^ visit	Week number												Total visits
		16	20	24	28	30	32	34	36	37	38	39	40	
Traditional prenatal care schedule (1=visit)	1	1	1	1	1	1	1	1	1	1	1	1	1	13
Babyscripts prenatal care schedule (0=no visit, 1=visit)	1	1	0	1	1	0	1	0	1	0	1	1	1	9

^a^OB: obstetric.

### Study Arms

Participants who were assigned to the experimental group were instructed to download the mobile app and set up the connected monitoring devices at the time of enrollment. Participants incurred no additional costs for the app or connected devices. Participants allocated to the control arm were not given access to the mobile app. The experimental group was also placed on an alternative prenatal care schedule of 8 expected visits as compared with the typical 12-14 expected visits in the control group (Table 1). The experimental visit schedule was based on the Department of Defense-Veterans Affairs Uncomplicated Pregnancy Guidelines [14]. For all participants, prenatal care met established guidelines for the management of uncomplicated pregnancies.

### Data Collection

Patient demographics were obtained by self-report at the time of enrollment. Detailed patient characteristics were also collected at the time of enrollment, including age, race, insurance status, and ethnicity. The primary outcome of the study was the number of in-person prenatal care visits as assessed by patient chart review. As a secondary outcome, all patients were evaluated for satisfaction with their prenatal care experience and pregnancy outcomes. (Multimedia Appendix 2) Patient satisfaction surveys were administered to participants at gestational weeks 16, 20, 25, 30, and 35 and 2 weeks postpartum. Satisfaction survey consisted of 16 questions using a 4-point Likert scale modified from the hospital consumer assessment of health care providers and systems survey instrument [15] In addition, 6 questions specific to the Babyscripts platform were submitted to participants in the experimental group. Participants who completed all 6 surveys and responded to all related questionnaires were compensated with a US$20 Amazon gift card at completion of the study. An additional secondary outcome was obtained via a chart review for significant clinical outcomes—preeclampsia, eclampsia, neonatal intensive care unit admissions, stillbirth, neonatal mortality, other serious outcome per investigator judgment, and route of delivery including cesarean delivery rate. Chart reviews were completed using trained abstractors with defined data collection sheets.

### Statistical Analysis

We compared outcomes between study arms using *t* tests for continuous outcomes and chi-square tests for dichotomous outcomes. For differences in the baseline characteristics between the 2 study groups, we used a hierarchical generalized logistic or linear regression model that includes an indicator for the study arm. We conducted descriptive heterogeneity of treatment effect analyses by age, gender, race/ethnicity, and highest attained maternal education level.

### Description of Mobile App

The Babyscripts app was designed with 2 major goals: (1) to deliver educational content via a mobile app and (2) to remotely monitor blood pressure and weight. The educational content was based upon ACOG standards and refined by a committee of 4 board-certified obstetricians at the GWU School of Medicine. Input from a variety of other stakeholders including patients, midwives, and administrators was also obtained. The mobile app sends educational content to the expectant mother at gestation-appropriate times throughout the pregnancy. This information encompasses material covering pregnancy progression; preexisting risk hazards such as alcohol intake, smoking, or drug abuse; advice to address these risk hazards; dietary and nutritional content; breastfeeding information; guidelines for appropriate weight gain; and warning signs for pregnancy complications. In addition, the app integrates with a Wi-Fi-connected scale and blood pressure cuff to provide both feedback and alerts depending on the readings. The alerts were created to provide early warnings to patients and providers about aberrant data points with the hope of providing early detection of hypertensive disorders of pregnancy and abnormal weight gain, indicating an increased risk of gestational diabetes, nutritional deficiency, or edema associated with preeclampsia.

## Results

### User Statistics

**Figure 1:**
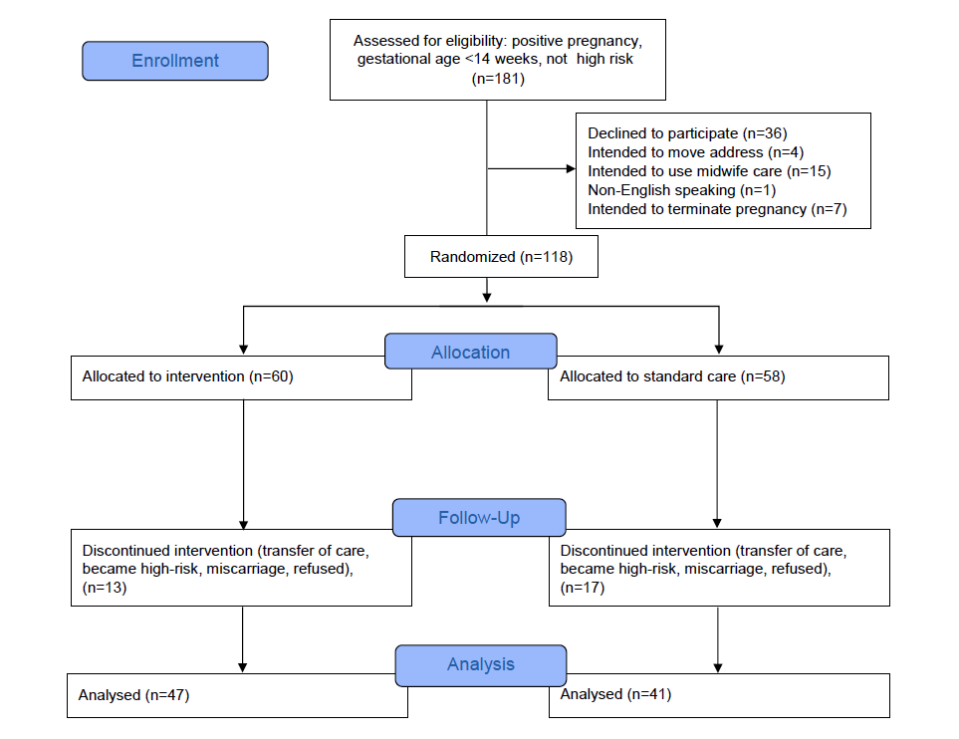
CONSORT flow diagram.

A total of 181 women met the inclusion criteria and were screened for enrollment. Of those, 118 met screening criteria and agreed to participate. Furthermore, 60 women were allocated to the experimental group and 58 women were allocated to the control group (Figure 1). Of those, 13 participants in the experimental group and 17 in the control group discontinued involvement in the study because they transferred care (n=11), developed high-risk characteristics (n=7), experienced a miscarriage (n=5), requested to no longer participate (n=2), or other reasons (n=5). Ultimately, 47 participants in the experimental group and 41 in the control group were retained in the study until completion and were analyzed. There was no significant difference in baseline characteristics (Table 2).

**Table 2:** Patient characteristics and number of visits per group.

Characteristics		Babyscripts (n=47)	Standard care (n=41)
Age at screening (years), mean (SD)		33.0 (3.3)	32.2 (3.2)
Body mass index at screening^a^, mean, kg/m2, (SD)		22.9 (3.2)	24.9 (4.0)
African-American, n (%)		14 (30)	13 (32)
Hispanic, n (%)		3 (6)	3 (7)
College graduate, n (%)		45 (96)	36 (88)
**Gravidity per patient, n (%)**			
	One	25 (53)	22 (53)
	Greater than one	22 (47)	19 (46)
**Parity per patient, n (%)**			
	Zero	30 (64)	25 (61)
	Greater than one	17 (36)	16 (39)
Number of in-person prenatal care visits^a^, mean (SD)		7.9 (1.8)	10.2 (1.8)

^a^*P*<.001.

### Evaluation Outcomes

**Figure 2:**
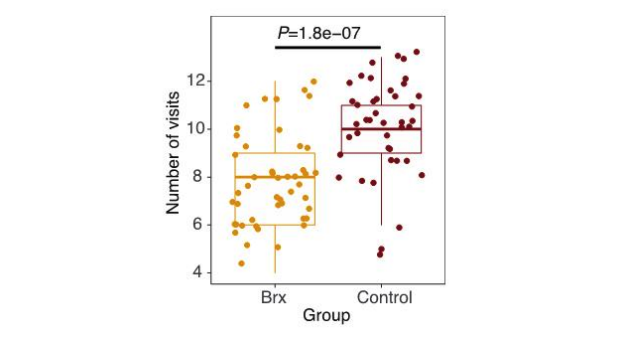
Total number of prenatal clinical visits per group. Babyscripts (Brx) versus controls. *P* value is based on Wilcoxon ranked-sum test.

**Figure 3:**
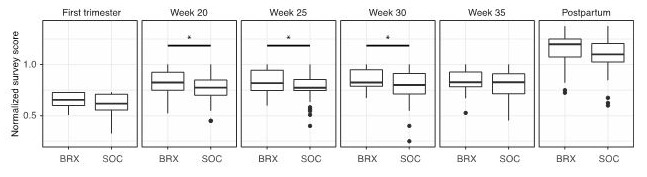
Survey data for patient satisfaction with overall prenatal care between Babyscripts (Brx) and control groups. For each time point, Brx and control patients are given a survey. The total numeric scores for all questions at that time point is calculated. All numeric scores are then normalized to 1 to standardize between different numbers of survey questions. SOC: standard of care.

The experimental group had significantly fewer prenatal care visits (7.9 visits) than patients in the control group (10.2 visits; *P*=.01 Table 2; Figure 2). Patient satisfaction measured over several intervals demonstrated no difference in satisfaction between the experimental and control group. Satisfaction scores were aggregated for all 16 questions that were asked to participants in both the control and experimental groups at each time point during gestation. For data visualization, the scores were normalized from 0 to 1 and then compared for statistical differences at each time point. Provider surveys demonstrated aggregate scores demonstrating highly perceived quality and satisfaction with the virtual care platform. There was no statistical difference in patient satisfaction (*P*>.05) or provider satisfaction (*P*>.05) in either group (Figure 3).

Although maternal and fetal outcomes were tracked, the study was not powered to demonstrate the effect of the virtual care platform on maternal or fetal outcomes. We identified 1 adverse fetal event of stillbirth at 38 weeks gestation. Investigation of the case revealed that the patient had an uncomplicated pregnancy and was compliant with the prenatal care schedule of the experimental group. Her obstetrician saw her in person within 3 days of the fetal demise at which time normal fetal movement and fetal heart tones were identified. Workup revealed a likely fetal-maternal hemorrhage unrelated to the study protocol.

## Discussion

### Principal Findings

Pregnant women represent a promising target for digital health apps. Unlike digital health apps that target chronically ill or elderly populations, pregnant women are a young and healthy population. A mobile health app that targets pregnant women may facilitate the integration of prenatal care into other aspects of their family and professional life. In addition, pregnancy is a unique period of life when healthy behaviors including exercise, diet, and sleep take on greater importance. As such, women are highly engaged with their health care decisions during pregnancy and may be more receptive to educational programs that can be delivered through a mobile health app. Finally, the majority of prenatal care visits are scheduled to exchange educational information with the patient or weight and blood pressure data with the provider. Both of these exchanges are especially amenable to communication via mobile technology or remote monitors. Ultimately, the app does not replace in-person visits but may replace some of the current activities that occur at each visit. If the app can communicate basic educational components of prenatal care, the in-person visits may allow for more individualized discussions.

As part of this study, several important elements emerged as critical to the success of a mobile prenatal care app. First, the initial assessment is critical to identifying high-risk versus normal-risk on initial assessment; second, accurate communication of patient data to the provider is necessary to assess early signs of pregnancy complications; third, educational information must be provided at the appropriate time during pregnancy; fourth, educational information should be targeted to individual patient (eg, not all women need regular reminders about the importance of smoking cessation); and fifth, a clear explanation that the role of the mobile app is to augment and not replace the obstetrician or midwife.

### Limitations

There are several limitations to our study. First, the use of a quasi-randomization scheme where participants were allocated by type of phone creates potential confounders, as it is possible that iOS users differ from non-iOS users. However, there was no significant difference in age, race, education level, gravity, or parity between the cohorts. We did not collect data on differences in household income, a possible confounder. The second risk concerns the possibility of contamination across groups. It is possible that by reducing visits in the experimental group, physicians were more likely to reduce the visits in all patients. However, given there was a statistically significant difference in visit schedule, this was unlikely to confound this cohort. Third, the mobile prenatal care app was prescribed as part of the reduced care schedule, and it is unknown if a reduced care schedule might have been effective without the mobile care app or with a different solution. Finally, we have limited information regarding emergency visits or phone calls that may have occurred outside of the chart review or the direct patient calls. It is possible that there were some therapeutic interactions missed in both the control and experimental group.

### Conclusions

In conclusion, satisfaction was unchanged and visits were reduced through the use of the prenatal mobile care app, Babyscripts. Future studies will look for predictors of adverse clinical outcomes in a variety of populations in hopes of mitigating risk of adverse events.

## Appendix

Multimedia Appendix 1 Excluded high-risk obstetrical conditions (n=84).

Multimedia Appendix 2 Patient Satisfaction Survey, weeks 14, 20, 25, 30, 35, and postpartum.

## Abbreviations

ACOG: American College of Obstetricians and Gynecologists
GWU: George Washington University
GYN: gynecology
IRB: Institutional Review Board
OB: obstetric
