# Supplementary material for: A Mobile Prenatal Care App to Reduce In-Person Visits: Prospective Controlled Trial
Source: JMIR Mhealth Uhealth. 2019 May 1;7(5):e10520. doi: 10.2196/10520 (PMC6658303; doi:10.2196/10520)
Supplement: Multimedia Appendix 1 [file mhealth_v7i5e10520_app1.pdf]

Alcoholism	1
BMI > 35	18
Cerclage	4
Coagulopathy	5
Diabetes	1
Endometriosis	1
h/o GDM	1
h/o HELLP	2
h/o Multiple Miscarriages	1
h/o Myomectomies	2
h/o Pre-term Birth	7
h/o Preeclampsia	6
Hypertensive	16
Hyperthyroid	1
Hypothyroid	3
IUGR	1
IVF Pregnancy	4
Mullerian Anomaly	5
Twin Births	8
Oligohydramnios	1
Placenta Previa	1
Placental Abruption	1
Rheumatoid Arthritis	1
Seizure Disorder	1
Severe Nausea/Vomiting	1
