# Supplementary material for: A Mobile Prenatal Care App to Reduce In-Person Visits: Prospective Controlled Trial
Source: JMIR Mhealth Uhealth. 2019 May 1;7(5):e10520. doi: 10.2196/10520 (PMC6658303; doi:10.2196/10520)
Supplement: Multimedia Appendix 2 [file mhealth_v7i5e10520_app2.pdf]

Satisfaction Measure	Agree/ Strongly Agree
I am satisfied with the prenatal care I have received.	
I feel connected to my healthcare provider.	
I am getting the care that I need when I need it.	
I have easy access to my healthcare provider's resources and educational materials.	
I feel knowledgeable about my pregnancy.	
I feel I am adopting healthy behaviors during my pregnancy.	
I feel that my healthcare provider knows how I am doing between my appointments.	
My healthcare provider and I are "partners" in my pregnancy.	
I feel knowledgeable about which type of exercise is safe to do during my pregnancy.	
I feel knowledgeable about healthy dietary habits during my pregnancy.	
I feel that I know what to expect during my second trimester.	
I feel comfortable knowing that my healthcare provider is monitoring my progress through Babyscripts between visits.	
It is convenient for me to weigh myself at least once weekly.	
It is convenient for me to measure my blood pressure at least once weekly.	
I feel comfortable sharing my data with my healthcare provider in between visits.	
Babyscripts delivers the right amount of information to me throughout my pregnancy.	
